# Oscillatory pattern of glycemic control in patients with diabetes mellitus

**DOI:** 10.1038/s41598-021-84822-5

**Published:** 2021-03-11

**Authors:** Manuel Vasquez-Muñoz, Alexis Arce-Alvarez, Magdalena von Igel, Carlos Veliz, Gonzalo Ruiz-Esquide, Rodrigo Ramirez-Campillo, Cristian Alvarez, Robinson Ramirez-Velez, Fernando A. Crespo, Mikel Izquierdo, Rodrigo Del Rio, David C. Andrade

**Affiliations:** 1 Clínica Santa María, Santiago, Chile; 2 Navarrabiomed, Complejo Hospitalario de Navarra (CHN), Universidad Pública de Navarra (UPNA), IdiSNA, Pamplona, Navarra Spain; 3 Escuela de Kinesiología, Facultad de Salud, Universidad Católica Silva Henríquez, Santiago, Chile; 4 Centro de Investigación en Fisiología del Ejercicio, Facultad de Ciencias, Universidad Mayor, Santiago, Chile; 5 Human Performance Laboratory, Quality of Life and Wellness Research Group, Department of Physical Activity Sciences, Universidad de Los Lagos, Osorno, Chile; 6 CIBER of Frailty and Healthy Aging (CIBERFES), Instituto de Salud Carlos III, Madrid, Spain; 7 DAiTA Lab, Facultad de Estudios Interdisciplinarios, Universidad Mayor, Santiago, Chile; 8 Laboratory of Cardiorespiratory Control, Department of Physiology, Pontificia Universidad Católica de Chile, Santiago, Chile; 9 Centro de Envejecimiento Y Regeneración (CARE), Pontificia Universidad Católica de Chile, Santiago, Chile; 10 Centro de Excelencia en Biomedicina de Magallanes (CEBIMA), Universidad de Magallanes, Punta Arenas, Chile; 11 Centro de Investigación en Fisiología Y Medicina de Altura (MedAlt), Facultad de Ciencias de la Salud, Universidad de Antofagasta, Av. Universidad de Antofagasta #02800, Antofagasta, Chile

**Keywords:** Type 1 diabetes, Type 2 diabetes, Type 1 diabetes

## Abstract

Daily glucose variability is higher in diabetic mellitus (DM) patients which has been related to the severity of the disease. However, it is unclear whether glycemic variability displays a specific pattern oscillation or if it is completely random. Thus, to determine glycemic variability pattern, we measured and analyzed continuous glucose monitoring (CGM) data, in control subjects and patients with DM type-1 (T1D). CGM data was assessed for 6 days (day: 08:00–20:00-h; and night: 20:00–08:00-h). Participants (n = 172; age = 18–80 years) were assigned to T1D (n = 144, females = 65) and Control (i.e., healthy; n = 28, females = 22) groups. Anthropometry, pharmacologic treatments, glycosylated hemoglobin (HbA1c) and years of evolution were determined. T1D females displayed a higher glycemia at 10:00–14:00-h vs. T1D males and Control females. DM patients displays mainly stationary oscillations (deterministic), with circadian rhythm characteristics. The glycemia oscillated between 2 and 6 days. The predictive model of glycemia showed that it is possible to predict hyper and hypoglycemia (R^2^ = 0.94 and 0.98, respectively) in DM patients independent of their etiology. Our data showed that glycemic variability had a specific oscillation pattern with circadian characteristics, with episodes of hypoglycemia and hyperglycemia at day phases, which could help therapeutic action for this population.

## Introduction

Diabetes mellitus (DM) is a significant public health problem, affecting 415 million adults, in addition to 318 million prediabetics^1^. Importantly, public spending associated with DM reached US$237 billion in 2017 in the US^2^. Moreover, DM is a significant cause of blindness, kidney failure, heart attacks, strokes, and lower limb amputation^3,4^. Considering the prevalence, poor prognosis and hazards associated with DM, it is essential to monitor, control, and stabilize the glycemia in this population. Along with this, it has been proposed that glycosylated hemoglobin (HbA1c) could predict the risk of long-term diabetes complications^3–6^. However, HbA1c allows only long-term metrics, limiting personalized therapy, particularly in DM type 1 patients (T1D). Compared to HbA1c, continuous glucose monitoring (CGM) allows real-time measurements of glucose, although it is feasible to assess hypoglycemia/hyperglycemia episodes and consequently glycemia variability over several days, weeks or even months^7,8^ However, whether the dynamic of glycemic fluctuation is exclusively represented by a specific pattern variability (stationary) or random oscillation (non-stationary) has not been completely explored. In fact, Kovatchev et al. (2016, 2017)^9,10^ indicated that the dynamic of glycemic fluctuation is more related to stationary signals rather than to random oscillation; however, it is no clear if a different pattern of oscillation occurs between patients, which could be relevant to different therapies applied in DM population.

Along to the aforementioned, glucose variability is of such relevance due that vascular complications occurrences in DM patients have been attributed to hyperglycemias and dysglycemias (higher and lower levels of glycemia during the day and night phases) events^9–12^. Additionally, despite that robust information exists regarding CGM data^7,8^, today there are no predictive models to extrapolate the moment and magnitude of hyper and hypoglycemia in DM patients. This is extremely relevant, considering that severe cardiovascular complications are related to dysglycemic events^11,12^. Thus, glycemia variability and its patterns may be considered a health issue; however, (i) whether a given specific pattern wavering represents glycemia oscillation and (ii) if it is possible, to predict severe glycemic events, is yet to be determined. Therefore, we hypothesized that glycemia control during the day and night phases accomplish a stationary pattern with characteristics of circadian rhythm oscillation, which is possible to modulate by a mathematical function.

## Results

### Baseline characteristics of T1D and control participants

**Figure 1:**
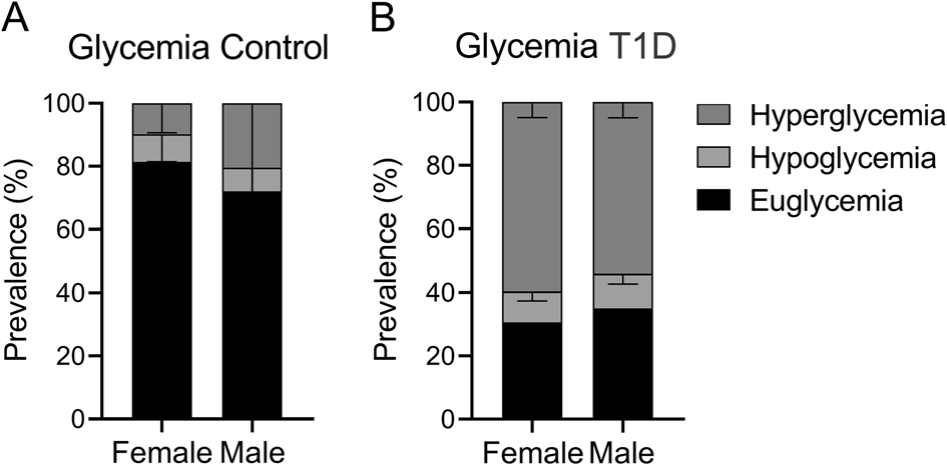
Prevalence of time spent in different glycemic states (hyperglycemic, hypoglycemic and euglycemic period). In females and males with diagnosis of T1D and Control group. One-way ANOVA, following Holm-Sidak posthoc.

Baseline characteristics are described in Table 1 and Supplementary Table S1. Age, HbA1c, body mass index (BMI), period of euglycemia, period of hyperglycemia episodes, and standard deviation (SD) of glucose were significantly different between Control compared to T1D patients (Supplementary Table S1, all *p* < 0.01). Females with T1D showed a significant decrease in body weight and height compared to their Control group (Table 1). Euglycemia and hyperglycemia period in females and males with T1D were significantly lower (*p* < 0.05) and higher (*p* < 0.05), respectively, compared to their sex-matched Control group (Fig. 1). The average daily units of ultra-rapid insulin were calculated for each group and there was no difference between groups (Male vs. female; from T1D group; Table1).

**Table 1:** Baseline characteristics of subjects with T1D and control groups, with subgroups of females and males.

	Control		T1D		*p* value	Distribution F (DFn, DFd)
	Females (n = 22; 78.57%)	Males (n = 6; 21.43%)	Females (n = 65; 45.14%)	Males (n = 79; 54.86%)		
Age (years)	40.17 ± 18.12	47.33 ± 19.58	34.68 ± 15.42	30.72 ± 14.41*	< 0.0001	(2, 181) = 62.33
Weight (kg)	71.04 ± 7.38	68.50 ± 7.01	63.49 ± 6.71^†^	69.54 ± 13.29	0.1785	(2, 181) = 1.740
Height (m)	169.09 ± 8.95	170.50 ± 8.57	157 ± 22^†^	170 ± 14	0.2723	(2, 181) = 1.310
BMI (kg/m^2^)	24.86 ± 2.00	24.23 ± 1.42	24.24 ± 2.48	23.74 ± 3.72	< 0.0001	(2, 181) = 21.70
Duration of diabetes (years)	–	–	29.48 ± 12.60	23.43 ± 11.10	0.0267	(1, 154) = 5.004
**Treatment (%)**						
Units of insulin per day (U/ml)	–	–	5.0 ± 1.51	5.2 ± 1.9	0.36	
Rapid acting insulin						
Aspart	–	–	n = 21 (33.82%)	n = 24 (30.30%)		
Lispro	–	–	n = 31 (47.06%)	n = 34(42.42%)		
Long-acting insulin						
Lantus	–	–	n = 47 (73.53%)	n = 55 (69.70%)		
Tresiba	–	–	n = 3 (4.41%)	n = 4 (4.55%)		
Toujeo	–	–	n = 1 (1.47%)	n = 1 (1.52%)		
Levemir	–	–	n = 11 (16.18%)	n = 25 (31.82%)		

Data are showed as mean ± standard deviation (SD). SD of glucose: standard deviation of glucose. %CV for glucose: percentage coefficient of 2 variation for glucose. Two ways ANOVA, following Holm-Sidak post hoc. Units of insulin per day (mean ± SD).

*p < 0.005, Males T1D versus Males Control.

^†^*p* < 0.005, Females T1D versus Females Control.

### Glycemic status and stationary/non-stationary glycemia variability pattern in females and males with T1D and controls

**Figure 2:**
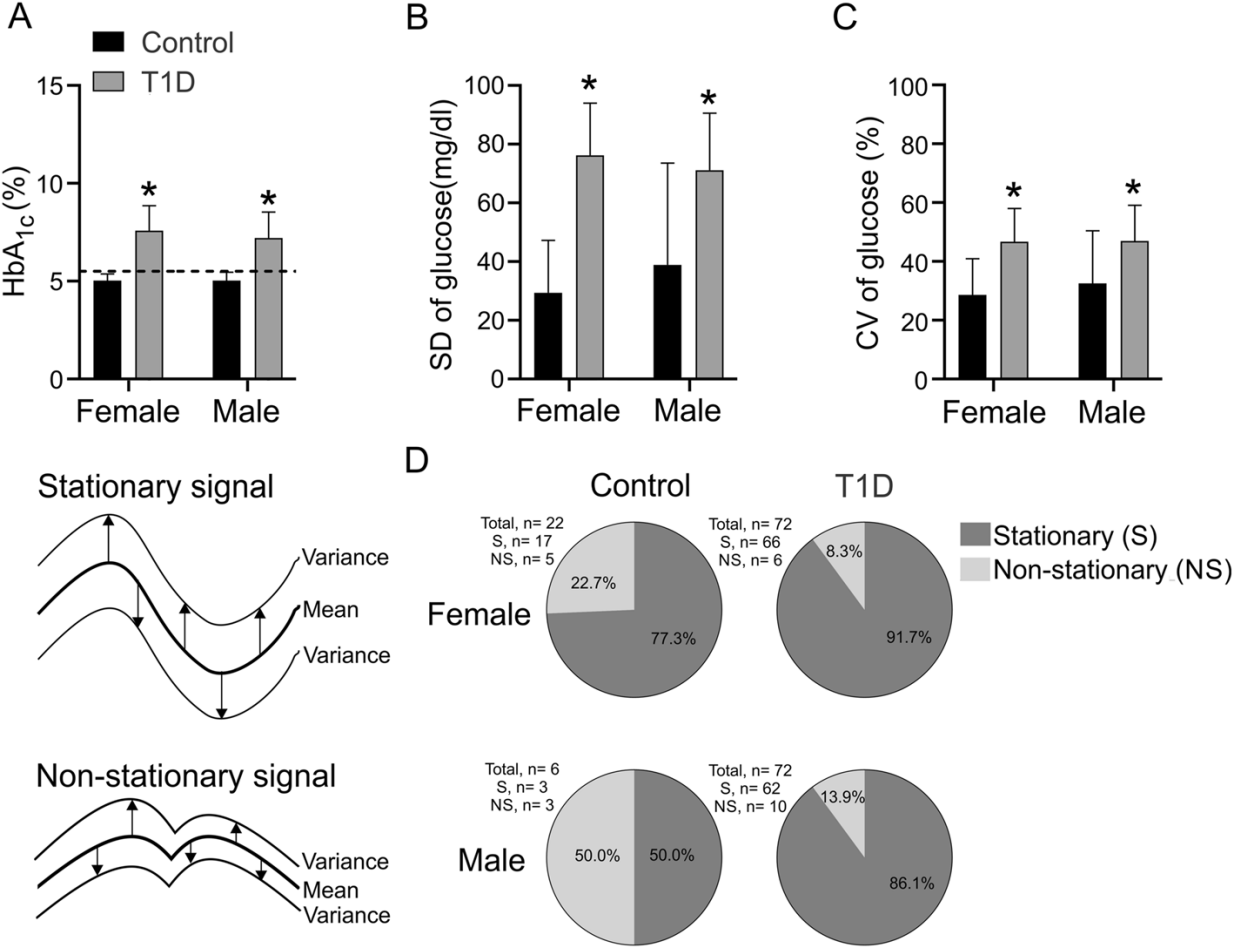
Glycosylated hemoglobin (HbA_1c_), glucose variability and prevalence of stationary and non-stationary glycemic oscillatory pattern in females and males with T1D and control participants. (**A**) HbA_1c_ is increased in T1D patients independent of sex. Indeed, T1D females and male patients showed a significant increase of HbA_1c_ compared to her/his matched Control group (Two ways ANOVA, following Holm-Sidak posthoc; F (DFn: 2, DFd: 181) = 32.43; *p* < 0.0001). (**B**) T1D females and males patients display an increase of standard deviation (SD) of glycemia compared to her/his matched Control group (Two ways ANOVA, following Holm-Sidak posthoc; F (DFn: 2, DFd: 180) = 35.30; *p* < 0.0001). (**C**) Compared to her matched Control group, T1D female subjects showed a significant increase of coefficient of variation (CV) of glycemia (Two ways ANOVA, following Holm-Sidak posthoc; F (DFn: 2, DFd: 180) = 15.89; *p* < 0.0001). Male with T1D showed a significant difference compared to his control group (Two ways ANOVA, following Holm-Sidak posthoc; F (DFn: 2, DFd: 180) = 15.89; *p* < 0.0001). (**D**) Left panel showed an example of stationary and non-stationary oscillatory signal. Stationary signal is characterized by a constant variance, and contrarily, non-stationary signal did not display a constant variance. Right panel showed the prevalence of stationary and non-stationary glycemic oscillatory pattern for 6 days of analysis. Stationary and non-stationary signals were analyzed by R Core Team (2020)^39^.

The HbA1c, calculated from CGM data, revealed that females and males with T1D displayed a significant increase of HbA1c compared to their respective sex-matched Control group (Fig. 2A, all *p* < 0.01). Regarding glucose variability data, the SD of glucose was significantly higher in males and females with T1D, compared to their respective sex-matched Control group (Fig. 2B, all *p* < 0.05). Females and males with T1D showed a significant increase in the coefficient of variation (CV) of glucose compared to their respective sex-matched Control group (Fig. 2C, *p* < 0.01).

To determine if glycemic variability accomplishes a deterministic or random oscillation, we evaluated if the signal displayed a stationary (deterministic) and non-stationary (random) behavior through the Dickey–Fuller test. Our analysis reveals that 91.7% and 86.1% of females and males with T1D, respectively, displayed a stationary glucose oscillatory pattern (Fig. 2D). In addition, 77.3% and 50.0% of Control females and males, respectively, showed a stationary pattern (Fig. 2D). These data suggests that participants accomplish for the most part stationary glycemia oscillatory pattern and not a random oscillation one.

### Glycemic oscillatory frequency in females and males with T1D

**Figure 3:**
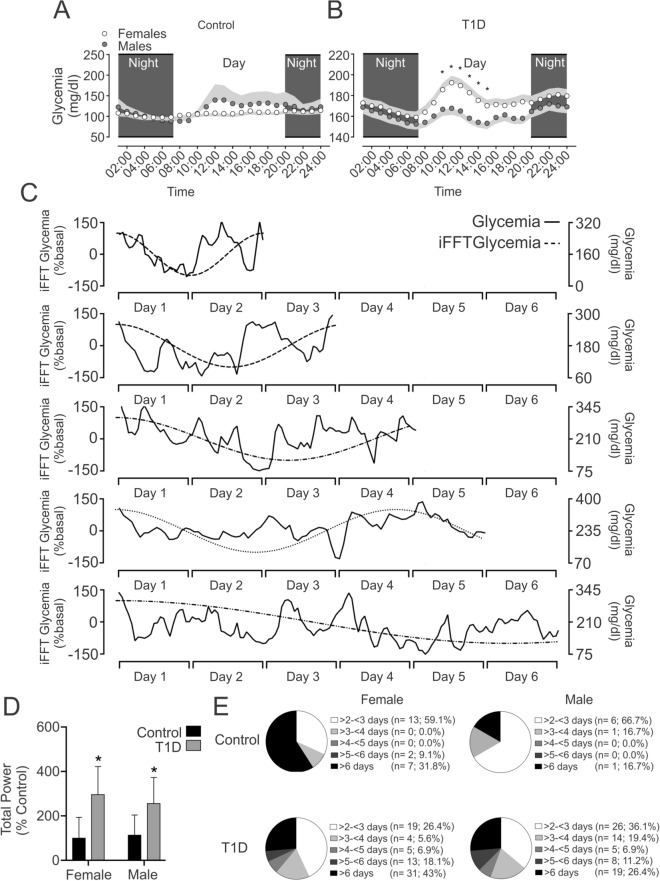
Circadian rhythm and oscillatory pattern of glycemia in patients T1D and control participant. (**A**) Females and males Control participant did not show significant difference on circadian rhythm of glycemia. (**B**) During day phase, females with T1D showed a significant difference compared to males with T1D patients between 10:00 to 15:00, without differences during night phases (Two ways ANOVA, following Fisher posthoc; F (DFn: 1, DFd: 133) = 3.51; *p* = 0.049). (**C**) Representative reconstruction from inverse Fast Fourier Transform (iFFT) of maximum energy glycemic oscillation and real oscillation of glycamia at 2, 3, 4 ,5 and 6 days. (**D**) Total power is increased in T1D patients independent of sex. Indeed, T1D females and males patients showed a significant increase of total power compared to her/his matched Control group (Two ways ANOVA, following Holm-Sidak posthoc; F (DFn: 2, DFd: 181) = 32.43; *p* < 0.0001). (**E**) Prevalence of maximum energy oscillation of glycemia in all experimental conditions. Note that in T1D patients (females and males) the maximum oscillation mainly is related to 2 to 3 days.

Females and males control group did not show significant differences during day and night phases (Fig. 3A). The CGM revealed that between 10:00–14:00 h, the female T1D patients displayed an increase of glycemia compared to T1D male patients (Fig. 3B, all *p* < 0.05).

The Fast Fourier Transform (FFT) approximation to determine the glycemic oscillatory pattern in DM patients showed that the total power of glycemia was significantly increased in T1D patients compared to the Control condition, regardless of sex (Fig. 3D; all *p* < 0.01). Moreover, the maximum glycemia oscillation varies from 2 to > 6 days (Fig. 3C,E). Female T1D patients displayed a prevalence for glycemia oscillation of 43.0%, 18.1%, 6.9%, 5.6%, and 26.4% on > 2 to < 3, > 3 to < 4, > 4 to < 5, > 5 to < 6, and > 6 days, respectively (Fig. 3F). Male T1D patients displayed a prevalence of 36.1%, 19.4%, 6.9%, 11.1%, and 26.4% on > 2 to < 3, > 3 to < 4, > 4 to < 5, > 5 to < 6, and > 6 days of glycemia oscillation, respectively (Fig. 3E).

Between 10:00–14:00 h, the female T1D patients displayed an increase of glycemia compared to the female Control group (4A, all *p* < 0.05). Males with T1D between 02:00–10:00 h, showed an increase of glycemia level compared to Control male participants (Fig. 5A; all *p* < 0.05).

**Figure 4:**
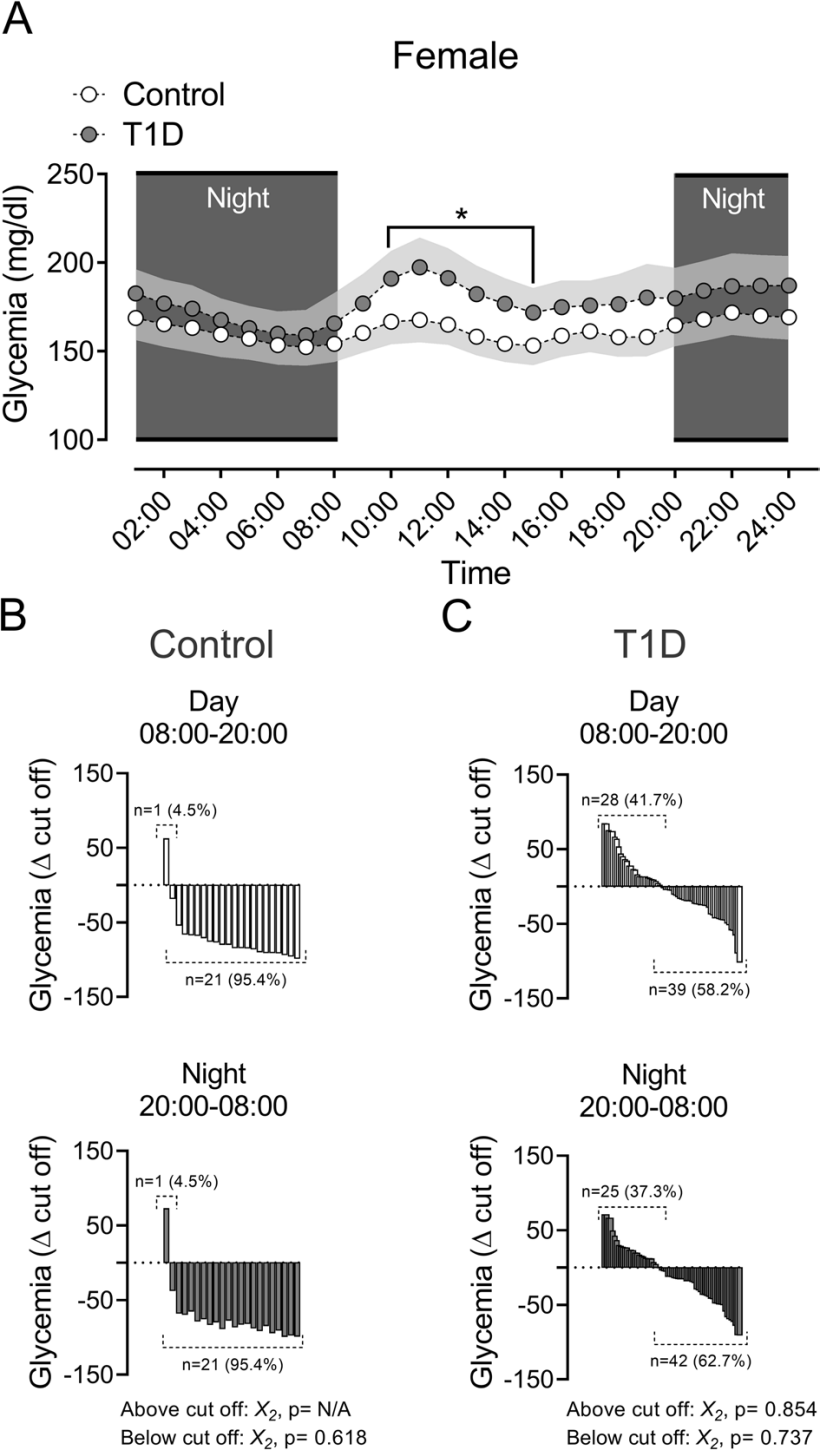
Circadian rhythm of glycemia and magnitude of hyperglycemia and euglycemia events during day and night phases in females T1D and control participant. (**A**) During day phase, females with T1D showed a significant difference compared to their matched control group between 10:00 to 15:00. (Two ways ANOVA, following Fisher posthoc; F (DFn: 2, DFd: 119) = 3.91; *p* = 0.022). (**B**,**C**) magnitude of hyperglycemias and euglycemia's during day and night phases, in Control and T1D female patients, respectively. There are no significant differences between all groups.

**Figure 5:**
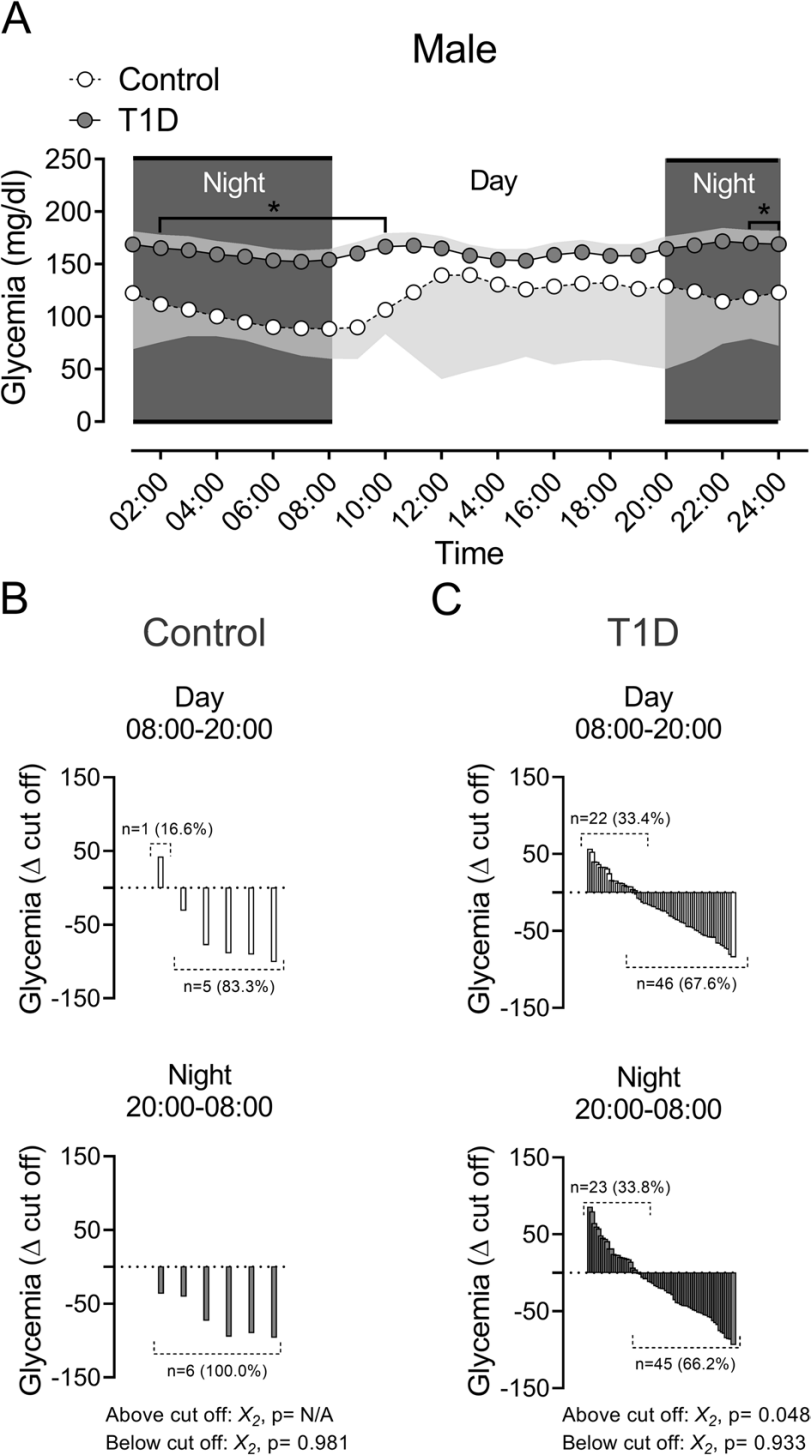
Circadian rhythm of glycemia and magnitude of hyperglycemia and euglycemia events during day and night phases in males T1D and control participant. (**A**) During day phase, T1D male patients showed a significant difference compared to their matched control group between 09:00 to 10:00. During night phases, males with T1D patients showed a significant difference compared to their matched control group between 21:00 to 08:00 (Two ways ANOVA, following Fisher posthoc; F (DFn: 2, DFd: 85) = 4.42; *p* = 0.015). (**B**,**C**) Magnitude of hyperglycemias and euglycemia's during day and night phases, in Control and T1D males' patients, respectively. Note that T1D male patients display a significant difference on hyperglycemias during day and night phases (Wilcoxon rank test, *p* = 0.048).

The interindividual variability of glycemia (hyperglycemia and euglycemia, from cut off: 180 mg/dL, accordingly to Miranda-Massari et al. 2016)^13^ during the day and night phases, revealed no significant differences between females Control and T1D participants (Fig. 4B,C). Regarding to males, T1D patients showed a significant difference in hyperglycemia magnitude between day and night phases (Fig. 5C; *p* < 0.05). Control participants didn´t show significant differences in interindividual variability during the whole experiment (Fig. 5B).

### The predictive model of hyperglycemia and hypoglycemia in DM patients.

**Figure 6:**
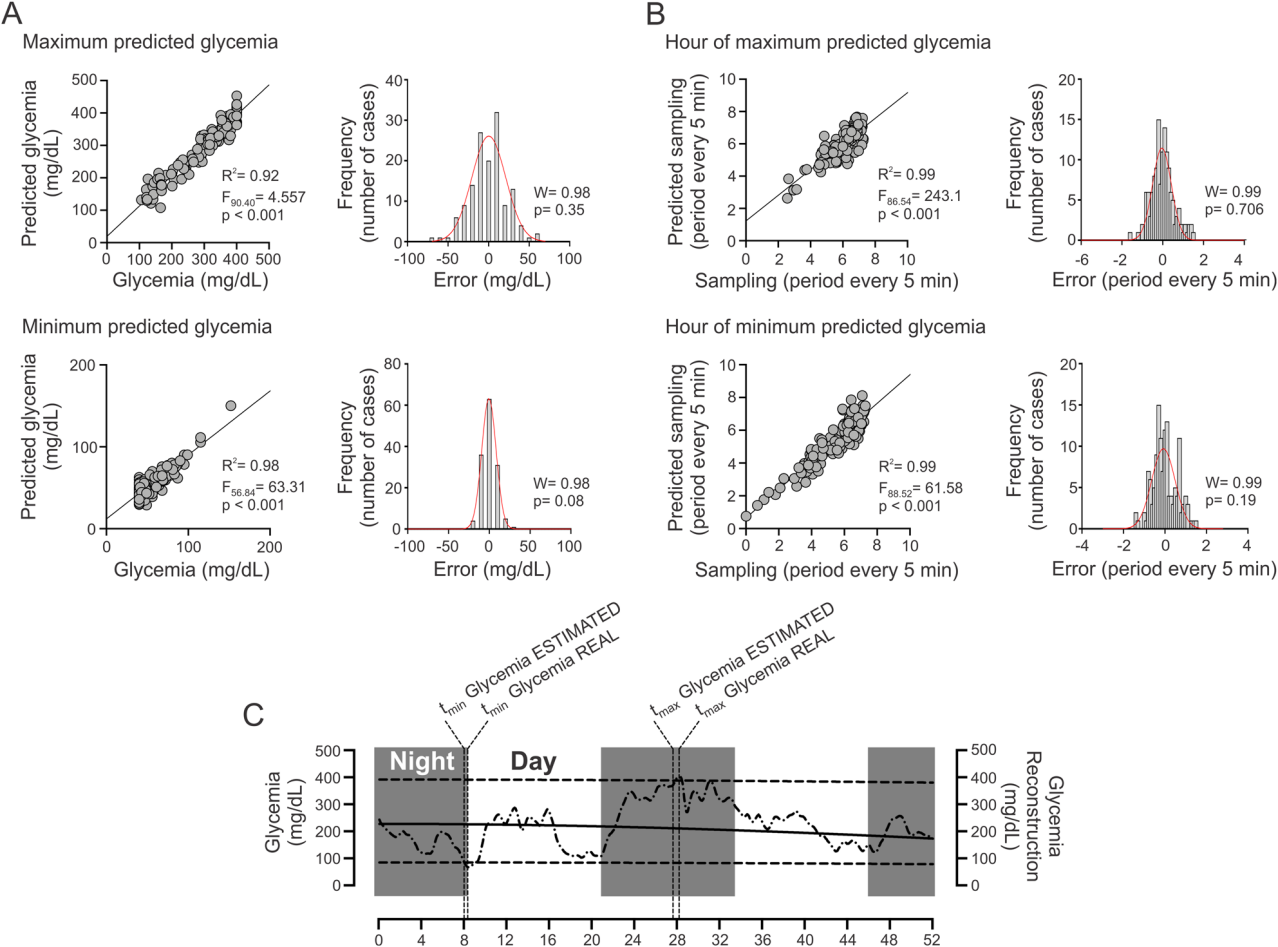
Predictive model of maximum and minimum glycemia and hour of maximum and minimum glycemia status in T1D patients. (**A**) (left panel) Scatterplot between real maximum glycemia and predicted maximum glycemia and between real minimum glycemia and predicted minimum glycemia. (right panel) Distribution of error of the model to maximum and minimum glycemia. The error was normal to maximum and minimum glycemia status. (**B**) (left panel) Hour of maximum and minimum predicted glycemia. (right panel) Distribution of error of the model to hour of maximum and minimum glycemia. The error was normal to both models. (**C**) Representative recoding of CGM and mean, minimum and maximum predicted glycemia of one DM patient. Horizontal segmented line represents minimum and maximum predicted glycemia. Vertical segmented line showed time of minimum (t_min_) and maximum (t_max_) real and predicted glycemia. Note that real and predicted glycemia are closely related.

The CGM data was used to create a predictive model to estimate hyperglycemia and hypoglycemia and additionally, to determine the exact time at which the hyperglycemia and hypoglycemia in DM patients was observed. Our data showed that it is possible to predict hyperglycemia’s (R^2^ = 0.92; F_90,40_ = 4.557; *p* < 0.001) and hypoglycemia’s in DM patients (R^2^ = 0.98; F_56,84_ = 63.31; *p* < 0.001) (Fig. 6A), which was independent of age. The error of our predictive model accomplished a normal distribution to hyperglycemia (W = 0.98; *p* = 0.35) and hypoglycemia (W = 0.98; *p* = 0.08) events (Fig. 6A). Along with these results, we found that it is possible to predict the time in which the hyperglycemia (R^2^ = 0.99; F_86,54_ = 243.1; *p* < 0.001) and hypoglycemia (R^2^ = 0.99; F_88,52_ = 61.58; *p* < 0.001) events occur (Fig. 6B). Regarding to the error of the model, our data revealed that the error to predict the time at which the events of hyperglycemia and hypoglycemia occurred, showed a normal distribution (W = 0.99; *p* = 0.706; and W = 0.99; *p* = 0.19, for hyperglycemia and hypoglycemia events, respectively) (Fig. 6B).

To evaluate the accuracy of our model, the recording of one patient was assessed (Fig. 6C). The data revealed that the real hyperglycemia was 395.9 mg/dL compared to the prediction, which was 388.3 mg/dL (Fig. 6C). Along with this, the real hypoglycemia was 70.16 mg/dL, and the predicted hypoglycemia was 68.0 mg/dL (Fig. 6C). On the other hand, the real hour at which hyperglycemia and hypoglycemia events were 28:13 and 08:22 h, respectively, compared to the predicted time of 27:38 and 08:01 h, hyperglycemia and hypoglycemia, respectively (Fig. 6C).

## Discussion

The main findings of the present study were: CGM data showed that (i) HbA1c and SD of glycemia were increased in DM patients in a sex-independent manner; (ii) glycemia variability displays a predominant stationary pattern (deterministic) in DM patients; (iii) half of the patients displayed a circadian rhythm of glycemia; (iv) regardless of sex, maximum oscillation was increased compared to control subjects (healthy); (v) females with T1D showed a specific rhythm pattern oscillation of glycemia, which was different between females and males; and (vi) CGM data allowed to construct a predictive model to determine the magnitude and time of hyperglycemia and hypoglycemia events. The results obtained could contribute to elaborate better control treatments and to prevent diabetes progression, which is characterized by dysglycemia events. The predictive model will allow to predict severe events related to hyper and hypoglycemia in DM patients.

Our data revealed that HbA1c was increased in T1D, independent of sex, which was similar to previous reports^14,15^. However, HbA1c only allowed the identification of sustained hyperglycemia but not completely related to dysglycemic events, which are related to the evolution of DM (i.e. long-standing patients) (SD of glycemia)^14–16^. Moreover, dysglycemia has been related to vascular complications and poor prognosis in DM patients^14–16^. Therefore, despite that HbA1c could be associated to the glycemic status in DM, it is not a robust measure to determine glycemic variability or the glycemic oscillatory pattern^16^. Nevertheless, it has been shown that, concomitant to an increase of HbA1c exists an enhanced SD of glycemia in T1D patients^15^. Our data demonstrates that SD and CV (glycemic variability estimators) were increased in DM patients, along with an increase of HbA1c, suggesting that HbA1c could be related to glycemic variability in DM patients. Notwithstanding, our data revealed that glycemic variability is mainly associated to a deterministic oscillation pattern rather than stochastic oscillations, suggesting that patients displayed a specific oscillatory arrangement and not a random oscillation, which is similar to previously described^9,10,17^ In addition, our data revealed that not all patients displayed the same oscillatory pattern. Indeed, our current results showed that DM patients could oscillate in phase (360°) between 2 and 6 days, which demonstrates that glycemic variability could oscillate outside of circadian rhythm. Therefore, our mathematical analysis based on FFT discriminates between circadian and non-circadian oscillators. However, our model is a closed loop, which could limit the extrapolation of our data. Similarly, the UVA/Padova system^17^, despite using machine learning and artificial intelligence, has reported similar limitations, associated to the long-term extrapolation of results. Consequently, future research should include longer follow-up periods and improve the current and future artificial pancreas system^17^ In addition, our data does not discard the possibility that glycemic oscillatory patterns could be secondary to poor glycemic control (increase of HbA1c). Therefore, patients with glycemic oscillatory patter should be the focus for future studies and determine if the glycemic control could contribute to generate a disruption of the circadian rhythm glycemic oscillation.

It has been demonstrated that blood glucose variability is an important marker related to disease severity^18^. However, if glycemic variability displays a stationary or non-stationary oscillatory pattern is under discussion^9,10^. A stationary oscillation is a signal characterized by stability over time, being shown a constant variance^19^. Our data demonstrates that DM patients displayed a predominantly stationary oscillation pattern of glycemic variability, determined through Dickey–Fuller test^20^, suggesting an interdependency between all data points. However, despite that Dickey–Fuller test showed that glycemic variability could be related to a stationary pattern (~ 90%), not all patients displayed this pattern. In fact, DM patients showed a little part of the population that showed a stochastic pattern (8.3 and 13.9%, Females and male T1D patients, respectively). Moreover, despite that ~ 90% of DM patients displayed stationary pattern, only ~ 30% displayed a circadian oscillation, which suggests that in these patients the possible therapies against decreasing glycemic variability, associated to circadian rhythm would not be very efficient.

It has been proposed that glycemic control could be related to hormone status^21^ as well as to carbohydrates intake^22^, which could modify the glycemic control in DM patients. Indeed, it has been found an association between age and the risk of suffering DM, concluding that late menopause reduces the risk of DM compared to early menopausal women^21–23^. Contrarily, a survey conducted in postmenopausal women showed that there exists a considerable increase in the risk of DM in over 50 years old non-obese women^24^. Despite that we could propose that carbohydrates intake could alter the circadian rhythm^25,26^ and consequently glycemic control. In our study the insulin administration was calculated in relation to g of carbohydrates intake and body weight (see Table 1 and Supplementary Table S1). Therefore, the non-stationary signal of DM patient is not related to carbohydrates intake. However, we cannot rule out the hormonal status, which should be resolved in future research.

On the other hand, a difference of diurnal hyperglycemia during day phase between female compared to male and their matched control group was found. This observation could be related to a reduced metabolic control in our patients^13–27^. Nevertheless, despite that, we determine the type, dose, and inoculation via of insulin that was delivered to the patients. It is essential to emphasize that we did not evaluate if patients consume glucocorticoids, which have hyperglycemic effects^28,29^. Thus, our data strongly suggests that these hours are pivotal in the glycemic control in female DM patients, which may improve future therapies in the DM population.

It has been described that not all patients display the same glycemic control during different states and treatments, which is called interindividual variability^30^. Accordingly, in the present study, we determined hyperglycemia’s (> 50% of 24 h, clinical cut-off > 180 mg/dL)^31^ and euglycemia’s during the day and night phases, between females and males with T1D. We did not find significant differences in inter individual variability between experimental groups, except in male T1D patients. Indeed, we found that hyperglycemia was more pronounced during the night phase compared to the day phase, which could be related to other pre-existing conditions in T1D male patients.

Considering that our data strongly suggest that glycemic variability displays a specific oscillation pattern, we tested a predictive model to determine hyper and hypoglycemia and the hour at which the events of hyper and hypoglycemia occurred. Our data reveals that we can predict these possible severe events in DM patients. Along with our data, it has been proposed that it is possible to predict hypoglycemia events and insulin doses delivered to DM patients^32,33^. It has also been shown that it is possible to predict the severity of the disease^34^. However, similar limitations were displayed in all predictive model, which are related to long-term (extrapolation) prediction^17^. Our model is no foreigner to this problem; therefore, even though we can predict hyper and hypoglycemia events, we cannot generate extrapolations for more than 6 days. In addition, our results could contribute to improve the automated insulin given system, adding new oscillatory pattern, possibly contributing to determine the hour and magnitude of hypo and hyperglycemia events in DM patients. The latter may help clinicians to determine and/or support medical decision based on CGM. Nevertheless, the possible role of oscillatory pattern on glycemic status (and vice versa), as well as the possible contribution to improve the current insulin deliver system should be addressed in future research.

## Strengths and limitations

From our perspective the greatest strength from our work was that glycemia signal displayed a stationary pattern (~ 90% total DM patients), but only ~ 30% accomplish a circadian rhythm oscillation. Also, we examined and compared characteristics of circadian rhythm pattern variability of glycemia in female and male patients with T1D. Finally, we created a new predictive model to determine hyper and hypoglycemia and the time when these events occur in T1D patients. Thus, our results could contribute to improve the current therapies and predictive models, which are based on machine learning and artificial intelligence^17^. In addition, our results could also contribute to improve the automated insulin deliver system, adding new oscillatory pattern. Also, our work could contribute to optimize public spending associated with this pathophysiological condition, since there are critical hours where hyperglycemia occurs.

As our study does not cover all the characteristics of the patients, it is essential to analyze in future research if this circadian oscillation pattern described by our study could be related to different medications, insulin treatments and planned physical exercise. Also, it is essential to emphasize that our study used a vast range of age in the experimental groups (18 to 80 years) which partially masked our results. Nevertheless, considering the complexity of the sample and the study aim (focusing on the glycemic variability, showing oscillation by circadian rhythm in control and type I DM patients), future research needs to elucidate this phenomenon in a more homogeneous sample.

Also, another limitation related to our study is the sample size in Control conditions, which contributed negatively to the interpretation of the data. However, despite that it is essential to stand out the number of participants, it is also relevant to emphasize that the CGM was developed for T1D patients, and a large part of the studies are developed for this group. Our study used a sample of 144 T1D participants; therefore, despite that sample size is an important limitation, our research applies a significant T1D participant.

## Conclusion

This study shows that through CGM data, patients with T1D exhibited mainly a glycemic variability with a specific oscillatory pattern that has specific circadian characteristics for each patient. Therefore, it is possible that the oscillatory pattern reveals sensitive time range, which could help the current system based on machine learning and/or artificial intelligence, including new oscillatory frequencies, which in turn could contribute to improve the automated insulin deliver system in this critical population.

## Materials and methods

### Patient population and ethical approved

Participants between 18 and 80 years of age were recruited either with diagnosis of T1D or healthy participants (Control). Those with DM had regular medical check-ups, and their treatments were fully described in clinical files. Patients were recruited from an Endocrinology and Diabetology service from Clínica Santa Maria, Santiago, Chile, between January 2015 and June 2019 (Supplementary Figure S1). Participants were excluded whether they had (i) gestational diabetes; (ii) < 6 days of continuous blood glucose monitoring; (iii) < 18 years-old; and (iv) female participants should not be in the first seven days of the follicular phase v) no clinical records. Patients were divided into two groups: T1D (n = 144, females = 65) and Control (healthy; n = 28, females = 22) group (Supplementary Table S1).

All methods and experimental protocols were carried out in accordance with the American Diabetes Association and in accordance with the Declaration of Helsinki (2013). In addition, all methods and experimental protocols were reviewed and approved by the Ethical Committee from Clínica Santa Maria, Santiago, Chile (approved #14). Written informed consent was obtained from each participant according to CIOMS Guideline # 4. All subjects were over 18 years old; therefore, the informed assent signed by a legal guardian was not necessary.

### CGM method and outcomes

During six consecutive days, a retrospective CGM system (Medtronic Inc., Northridge, CA) for subcutaneous interstitial glucose monitoring was used. This electronic device was inserted subcutaneously in the non-dominant arm, and removed after six days, under sterile conditions. All CGM recordings were performed blind with the Medtronic MiniMed iPro2 (iPro2 digital recorder) with an Enlite sensor (Medtronic Inc., Northridge, CA). Data was manually registered to calibrate the sensor data. The sample rate of the sensor was 0.003 Hz (one point every 5 min, 288 daily measurements). When a sensor failed, the missed data from recording was not replaced by interpolation or mean calculation. However, for analyses purposes, measurements obtained after each hour were averaged, generating 24 data points every day, for six days. In addition, to fast Fourier transform analysis, the missed data was replaced by the minimum energy oscillation reconstruction, as previously described^35^. The reports were analyzed individually to find calibration errors. Finally, the data was exported in CSV format. The CGM system determines interstitial glucose using a sensor with glucose oxidase, an enzyme that catalyzes the electrochemical reaction between glucose and oxygen, obtaining an electric current in nanoamps, wirelessly transmitted to the CGM receiver^36^. A calibration algorithm was used for the CGM system according to the manufacturer instructions. From continuous recordings, the HbA1c was determined according to the following formula: HbA1c % = (Avg glucose + 46.7)/28.7; after HbA1c mmol/mol = (10.93 × HbA1c %) − 23.5^36^. Where Avg glucose is the arithmetic mean calculated from all data points from CGM. All these calculations were performed with CareLink iPro software (version 2.2.005, Northridge CA, USA). Glycemia was reported as mg/dL.

After six days of CGM, glucose variability parameters were calculated. SD and CV were used as glucose variability outcomes^37^. To determine CV, the SD (from 6 days) was divided by the arithmetic mean (from 6 days) of the corresponding glucose reading. Written instructions regarding food consumption hours were provided. Food consumption times included breakfast (07:00–10:00 h), lunch (12:30–15:00 h), dinner (19:00–22:00 h) and post-dinner (22:30–00:00 h). The suggested caloric intake for patients with DM was prescribed according to the American Association of Clinical Endocrinologists and the American Diabetes Association recommendations. Carbohydrates, protein, and fat represented between 45–65%, 15–20% and < 30% of total daily energy intake, respectively. Fiber intake between 25–50 g/day was included. This recommendation was given to each patient advised by a certified nutritionist^38^. To determine the possible effects of pharmacological treatments in all experimental groups, we assessed the prevalence of the main medications that each patient received, taking into consideration that patients follow a scheme delivered by the diabetes unit. The DM patients administrated standard insulin before breakfast and the ultrafast insulin before every food intake. Patient education was conducted by qualified diabetes nurse educators and nutritionists from the Diabetologia Clinica Santa Maria team.

The treatment was divided into three groups: rapid-acting insulin and long-acting insulin, represented with “n” and % in each experimental group the patients did not receive any other medications that could affect glycemic control (See Table 1 and Supplementary Table S1). The insulin was administrated by a calculated bolus (relative to g of consumed carbohydrates and body mass) before each meal. The dosage is showed in Table 1 and Supplementary Table S1. The subjects were strongly advised to not engage or perform any physical exercise, other than their regular daily work-home activities; however, the daily activities were not restricted.

### Anthropometric variables and medical history

Every patient, who was adherent to the present research, underwent a physical examination that include determination of age (years), body mass (kg), height (m) and body mass index (BMI; kg·m^-2^). After that, their medical history was assessed, including treatment type and years with diabetes. The prevalence of euglycemia, hyperglycemia and hypoglycemia were determined in all experimental groups, through CGM.

### Stationary/non-stationary CGM analysis

To determine the stationary and non-stationary pattern of glucose variability from the time series provided by the CGM system, the Dickey–Fuller test was used as previously described^20^. The Dickey–Fuller test, which is a nonlinear estimation, assumes that the data has interdependency with the previous data point (delay time). To determine the stationary and non-stationary pattern, the following formula was used: yt = pyt − 1 + ut. Where y is glucose data, t is the time, and *p* is a constant coefficient related to autoregressive analysis^20^. A stationary variable was defined as a variable without a significant (*p* > 0.05) change in variance across all time. Contrarily, a non-stationary variable was defined as a variable with a significant (*p* < 0.05) change in variance across time. The analysis was performed using R Core Team (2020)^39^.

### Glycemic oscillatory pattern

The circadian rhythm was determined in all patients through the Fast Fourier Transform (FFT) algorithm, according to previous research^40^. For the current data structure, every glycemic signal corresponded to 1,440 data points, and missing data in the signal was replaced by the minimum energy oscillation reconstruction^35^. To obtain different frequencies of the cycles, the FFT algorithm was applied^41^, using the functions available from the FFT module in the NUMPY package (Python Anaconda 3.6.6, 64 bits version)^42^. From different frequencies, function pondered, and the power spectral densities of each pondered were obtained. Afterwards, the inverse FFT (iFFT) pondered was used to verify the quality of the estimation. Then, the frequencies with the highest power were selected (over 15,000 a.u.) according to energy weight signal^35^. The total power of the signal and the frequency at maximum power spectral density (PSD) were plotted. To determine the prevalence of different oscillatory pattern in all patients, the data was divided on > 2 to < 3; > 3 to < 4; > 4 to < 5; > 5 to < 6; and > 6 days to maximum oscillation. This analysis was performed using Python Anaconda 3.6.6, 64 bits version (Python Software Foundation, Amsterdam, Netherlands)^42^.

### The predictive model of glycemia

Our predictive model was the convergence of several successive steps to find the best linear models which display a higher and robust adjustment^42,43^. Thus, our algorithm was applied as follow: (i) natural logarithm transformed was applied to the time of the minimum and maximum glycemia; (ii) correlations were made between the variables of minimum and maximum glycemia and minimum and maximum glycemic time with all FFT weights (see “Glycemic oscillatory pattern” section). Where the first 140 data, for adjustment, are chosen at random. The operation was repeated at least 40 times per model to identify points which could generate problems in more than one adjustment model. Thirty-three data points that had difficulties in their adjustment behavior were taken from the total data, probably due to missing data points. With the remaining data, the final model was calculated. The Akaike criterion was applied to select the best model. Then the non-significant variables were eliminated, one by one, leaving only those variables that were significant for a level < 0.05 and with the adjusted models we proceeded to see in one case. This analysis was performed using the Python software version 3.8.1 (Python Software Foundation, Amsterdam, Netherlands).

### Statistical analyses

Data is expressed as mean ± SD or 95% confidence interval (glycemic oscillatory pattern data). All data was subjected to normality (Shapiro–Wilk) and homoscedasticity (Levene) testing. Data was evaluated using a 2 (control and T1D) × 2 (female-male) analysis of variance (ANOVA two way), followed by Holm-Sidak posthoc analysis according to the data structure. Non-parametric variables were evaluated using Kruskal–Wallis analysis followed by Dunn´s posthoc test. To determine the magnitude of hyperglycemia (clinical cut-off > 180 mg/dL)^31^ and euglycemia between female and male, during the day and night phases, the Mann–Whitney test was used. *p* < 0.05 was considered statistically significant. All analyses were performed with GraphPad Prism 9.0.1 (La Jolla, CA, USA) and R Core Team (2020)^39^.

## Supplementary Information

Supplementary Information
