# Supplementary material for: Oscillatory pattern of glycemic control in patients with diabetes mellitus
Source: Sci Rep. 2021 Mar 11;11:5789. doi: 10.1038/s41598-021-84822-5 (PMC7970978; doi:10.1038/s41598-021-84822-5)
Supplement: Supplementary file 1 — Supplementary Information [file 41598_2021_84822_MOESM1_ESM.pdf]

OSCILLATORY PATTERN OF GLYCEMIC CONTROL IN PATIENTS WITH DIABETES MELLITUS

**Manuel Vasquez-Muñoz[1,2]; **Alexis Arce-Alvarez[2,3]; Magdalena von Igel[4]; Carlos Veliz[4]; Gonzalo Ruiz-Esquide[1]; Rodrigo Ramirez-Campillo[5]; Cristian Alvarez[5]; Robinson Ramirez-Velez[2,6]; Fernando A. Crespo[7]; Mikel Izquierdo[2,6]; Rodrigo Del Rio[8,9,10]; *David C. Andrade[8,11].

[1]Clínica Santa María, Santiago, Chile; [2]Navarrabiomed, Complejo Hospitalario de Navarra (CHN), Universidad Pública de Navarra (UPNA), IdiSNA, Pamplona, Navarra, Spain; [3]Escuela de Kinesiología, Facultad de Salud, Universidad Católica Silva Henríquez, Santiago, Chile; [4]Escuela de Medicina y Escuela de Postgrado, Facultad de Ciencias, Universidad Mayor, Santiago, Chile; [5]Human Performance Laboratory, Quality of Life and Wellness Research Group. Department of Physical Activity Sciences. Universidad de Los Lagos. Osorno, Chile; [6]CIBER of Frailty and Healthy Aging (CIBERFES), Instituto de Salud Carlos III, Madrid, Spain; [7]DAiTA Lab, Facultad de Estudios Interdisciplinarios, Universidad Mayor, Chile; [8]Laboratory of Cardiorespiratory Control, Department of Physiology, Pontificia Universidad Católica de Chile, Santiago, Chile; [9]Centro de Envejecimiento y Regeneración (CARE), Pontificia Universidad Católica de Chile, Santiago, Chile; [10]Centro de Excelencia en Biomedicina de Magallanes (CEBIMA), Universidad de Magallanes, Punta Arenas, Chile; [11]Centro de Investigación en Fisiología y Medicina de Altura (MedAlt), Facultad de Ciencias de la Salud, Universidad de Antofagasta, Antofagasta, Chile

Running head: glycemia variability and diabetes mellitus

Disclosure statement: the authors have nothing to disclosure.

**Both authors contributed equally to this work.

*Corresponding author: David C. Andrade, Ph.D.
Centro de Investigación en Fisiología y Medicina de Altura (MedAlt)
Facultad de Ciencias de la Salud
Universidad de Antofagasta
Av. Universidad de Antofagasta #02800
Tel: +562 25189702
E-mail: david.andrade@uantof.cl
dcandrade@uc.cl

Supplementary Figure S1

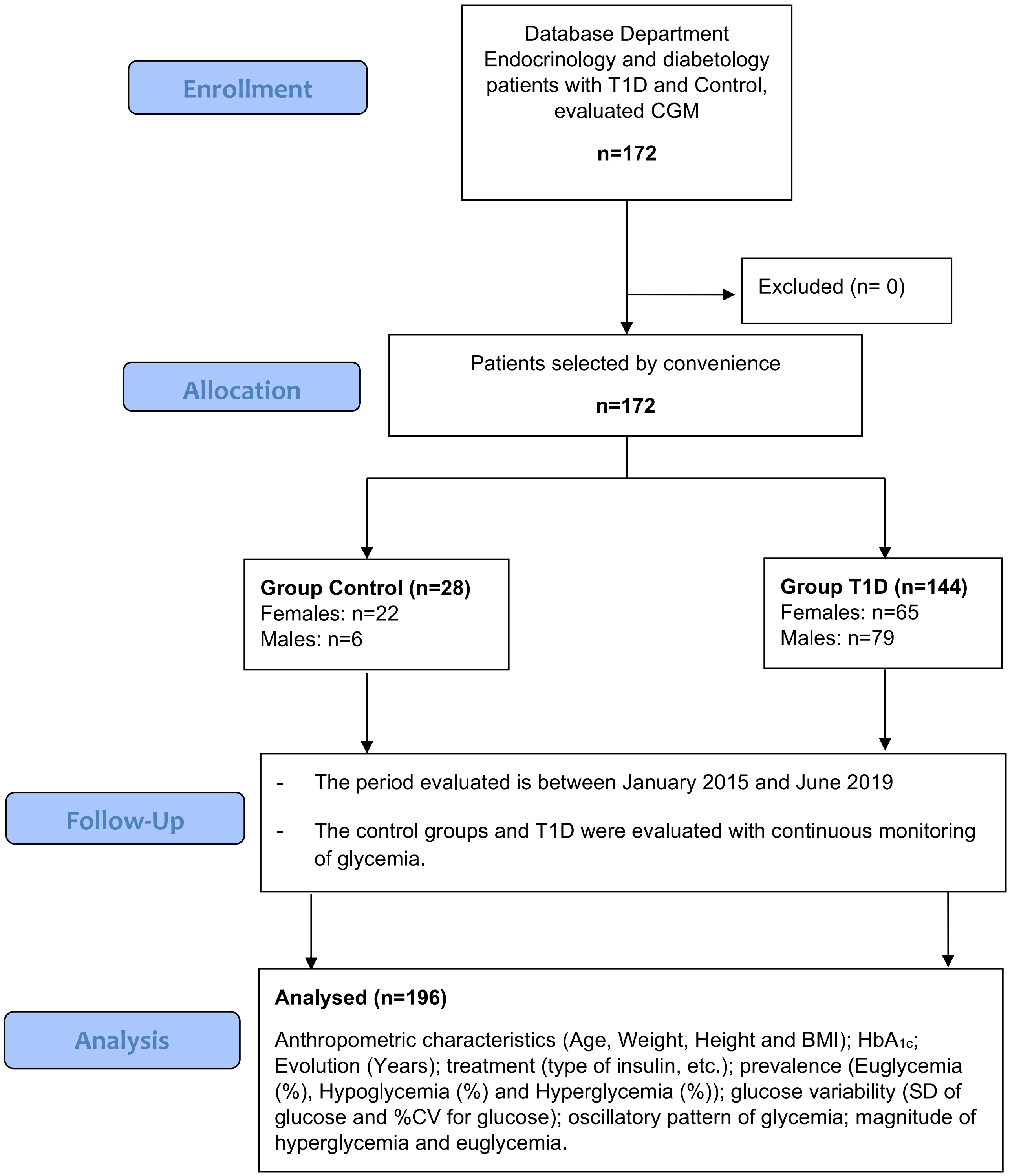

Supplementary Figure S1. Flow diagram Study Design. T1D: diabetes mellitus type I; CGM: continuous glucose monitoring; SD: standard deviation; and CV: coefficient of variation.

Table S1. Baseline characteristics of subjects with T1D and control groups.

	Control n = 28 (14.43%)	T1D n = 144 (74.22%)	P-value
Age (years)	41.65±18.3	32.67±14.99*	0.0034
Weight (kg)	70.52±7.26	66.56±10.96	0.4525
Height (m)	1.64±0.29	1.64±0.19	0.7729
BMI (kg/m-2)	24.73±1.88	23.99±3.16	0.0052
HbA1c (%)	5.03±0.34	8.89±8.47*	0.0025
Duration of diabetes (years)	---------------	26.41±12.20	0.1964
**Treatment (%)**			
Units of insulin per day (U/ml)	--------------	5.1±1.7	--------
Rapid Acting Insulin			
Aspart	---------------	n=45 (31.25%)	
Lispro	---------------	n=65 (45.13%)	
Long Acting Insulin			
Lantus	---------------	n=102 (70.83%)	
Tresiba	---------------	n=7 (4.86%)	
Toujeo	---------------	n=2 (1.38%)	
Levemir	---------------	n=36 (25%)	
Insulatard	---------------	n=102 (70.83%)	
Normal (%)	83.51±24.92	32.85±16.55*	0.0024
Hypoglycemia (%)	4.37±6.57	10.67±12.77	0.2126
Hyperglycemia (%)	2.79±3.25	57.11±19.75*	0.0006
SD of glucose	31.42±21.99	73.67±18.69*	0.0074
%CV for glucose	29.47±13.41	46.83±11.69	0.0921

Data are showed as mean ± standard deviation (SD). BMI: body mass index; HbA_{1c}: glycosylate hemoglobin; CV: coefficient of variation (relative). Unpaired T-test *, p<0.05, vs. Control.
